# Inequities in consistent condom use among sexually experienced undergraduates in mainland China: implications for planning interventions

**DOI:** 10.1186/s12889-019-7435-4

**Published:** 2019-08-30

**Authors:** Fang Ruan, Guochen Fu, Yongfu Yan, Yajie Li, Yulin Shi, Lan Luo, Xin Li, Bolun Zhang, Qinxin Gong, Zihan Fu, Yuhang Gan, Mengge Pan, Yusi Liu, Jihong Zhan, Junfang Wang

**Affiliations:** 1 Department of Preventive Medicine, School of Basic Medical Sciences, Hubei University of Science and Technology, No.88 Xianning Avenue, Xianning City, 437100 Hubei Province China; 2 National Demonstration Center for Experimental General Medicine Education of Hubei University of Science and Technology, No.88 Xianning Avenue, Xianning City, 437100 Hubei Province China

**Keywords:** Hierarchical logistic regression, Andersen’s behavioral model, Consistent condom use, Undergraduates, China

## Abstract

**Background:**

Since pre-exposure prophylaxis (PrEP) is mainly prescribed to high-risk uninfected individuals, consistent condom use (CCU) continues to be recommended as an inexpensive, feasible, practical and acceptable way to prevent the general population from acquiring and transmitting HIV through sexual intercourse. The objective of this cross-sectional study was to compare the relative importance of various determinants of CCU among sexually experienced undergraduates in mainland China so as to assess and subsequently to suggest ways to eliminate inequities in its use.

**Method:**

From September 10, 2018, to January 9, 2019, an anonymous self-administered online questionnaire was voluntarily completed by 12,750 participants distributed across 30 provinces in mainland China (except for Tibet). The present analysis was restricted to 2054 sexually experienced undergraduates. Pearson’s chi-square test and Logistic regression models were chosen to analyze the factors associated with CCU.

**Results:**

The overall rate of CCU was 61.3% [95% confidence interval (CI) = 59.2–63.4%]. CCU was inequitably distributed since enabling factors exerted greater effects than predisposing and need variables. Compared with heterosexual men, heterosexual women [adjusted odds ratio (AOR) = 0.78, 95% confidence interval (CI):0.64–0.96)], non-heterosexuals men (AOR = 0.64, 95% CI:0.45–0.92) and women (AOR = 0.68, 95% CI:0.47–0.99) were less prone to using condoms consistently. Those with more resources [i.e., higher levels of self- efficacy for condom use (AOR = 2.86, 95% CI:2.35–3.49) and being knowledgeable of the national AIDS policy (AOR = 1.50, 95% CI:1.23–1.82)], and those with lower need for condoms [i.e., late initiation of sexual activity (AOR = 1.34, 95% CI:1.09–1.64) and single sexual partner (AOR = 1.68,95% CI:1.21–2.33)] were more likely to be consistent condom users.

**Conclusions:**

In order to increase consistency of condom use and simultaneously reduce the remaining inequities, a comprehensive intervention measure should be taken to target heterosexual women, non-heterosexual men and women, and those with higher need for condoms, improve their condom use self- efficacy and raise their awareness of the national AIDS policy.

## Background

A tolerant attitude toward premarital sex and risky sexual behaviors are increasing the number of annually reported new cases of HIV infection among Chinese young students [1, 2]. According to the most recent figures available from the national HIV surveillance system, 527 young students aged 15–24 years were reported to be infected with HIV in 2008, and then the number reached 3236 in 2015. During the period between 2011 and 2015, the annual growth rate of new infections stood at a stunning 35%, with 65% of infections occurring in college students between18 and 22 years of age. Although the number of HIV diagnoses declined in 2016 and 2017, more than 3000 new cases were still identified each year. The predominant mode of HIV transmission among Chinese young students is through sexual intercourse, especially through male-to-male sexual contact, accounting for up to 81.6% of new HIV infections in 2014 [3]. Therefore, undergraduate college students, who are typically between the ages of 18 and 22 years in China, are vulnerable to contracting HIV as soon as they initiate sexual activity [2].

Consistent condom use (CCU) continues to be recommended as an inexpensive, feasible, practical and acceptable way to prevent the general population (e.g., college students) from acquiring and transmitting HIV through sexual intercourse, because pre-exposure prophylaxis (PrEP) is mainly prescribed to high-risk uninfected individuals such as commercial sex workers, men who have sex with men (MSM), and drug abusers [4], and also because PrEP alone does not protect against other sexually transmitted diseases (STDs) and unintended pregnancies [5].

In China, previous studies about condom use focused predominantly on most-at- risk populations such as female sex workers and their clients, STD clinic attendees, MSM, drug abusers, people living with HIV (PLHIV), and rural-to-urban migrants. In recent years, due to the vulnerability of unmarried sexually active college students to unintended pregnancy, HIV infection, and other STDs [2], a growing body of research has examined the frequency of condom use and its associated factors to develop appropriate interventions for this vulnerable population. It is noted that the number of studies examining condom use among college students in China is limited to six studies [6–11] and five quantitative surveys [6–10] suggest condom use is relatively low, ranging between 28% [9] and 68% [6], except for one qualitative survey [11]. Furthermore, these studies also indicated that condom use was associated with gender [7, 9], education backgrounds of parents [9], attitudes towards condom use [7, 8, 10, 11], condom use self-efficacy [7, 8, 11], partners numbers [9, 10], condom use at sexual debut [9], condom unavailable when having sex [10], and lack of sexual education from school and families [7, 11].

Currently, the Health Department of the Chinese Government is continuing its free condom initiative, with the goal of increasing the frequency of condom use and ensuring equity of access to condom use. Thus, it remains a question whether consistent use of condoms as one type of preventive services or preventive behaviors is equitably distributed. As described above, previous research has identified a set of variables associated with consistent condom use among college students in China. However, most of these associations have been explored with the Chi-square test or/ and through non-hierarchical logistic regression models, and few methodologically rigorous studies have systematically assessed inequities in consistent use of condoms with the guidance of explicit theoretical framework.

## Conceptual framework

In order to assess the magnitude of inequities and their determinants in consistent condom use and how these inequities might be narrowed or even eliminated, the Andersen’s behavioral model (ABM), which assumes a sequence of predisposing, enabling and need variables contributing to an individual’s health service use and health behavior adoption [12], was chosen as the conceptual framework of this study. The theoretical model, initially developed in the late 1960′ and experienced several rounds of revisions, has been successfully used to guide numerous studies on health service use and health behavior adoption, due to its diversity in the conceptualization and measurement of its components and also because of its capability to measure the presence of inequities and suggest ways to achieve equity of access to health services.

Factors predisposing individuals to use condoms include social-demographic characteristics {e.g., age [13], gender [7, 9, 14], sexual orientation [14, 15], race [13–15], marital status [13], education level [13, 15], education backgrounds of parents [9]}, and attitudinal-belief variables such as HIV-related knowledge [8, 13] and attitudes towards condom use [7, 8, 10, 11, 14, 17]. Factors inhibiting and promoting use of condoms (i.e., enabling factors) can be measured with multiple indicators such as their own personal resources {e.g., income [15] and self-efficacy of condom use [7, 8, 11, 14, 16, 17]} and the availability of health services in their community of residence such as condom available when having sex [10] and exposure to a community-level HIV prevention intervention [13, 16]. Need factors, representing the most immediate cause of condom use, comprise the objective and professional evaluation of need {i.e., risky sexual behaviors [27] such as having more sexual partners [2, 9, 10, 14], having casual sex partners [2], and early initiation of sexual activity [2, 21]} and the subjective assessment of need such as self-perceived risk of HIV infection [16], which is linked to an individual’s sexual history such as more frequent multiple partnerships [18]. Condom use is equitably distributed if it is primarily determined by need factors, while inequity occurs when use depends largely on enabling variables. “Mutability” reflects the extent that a given factor can be changed. Generally, social-demographic variables and need factors are not easily changed, while enabling variables and attitudinal-belief variables are relatively mutable [12].

**Fig. 1:**
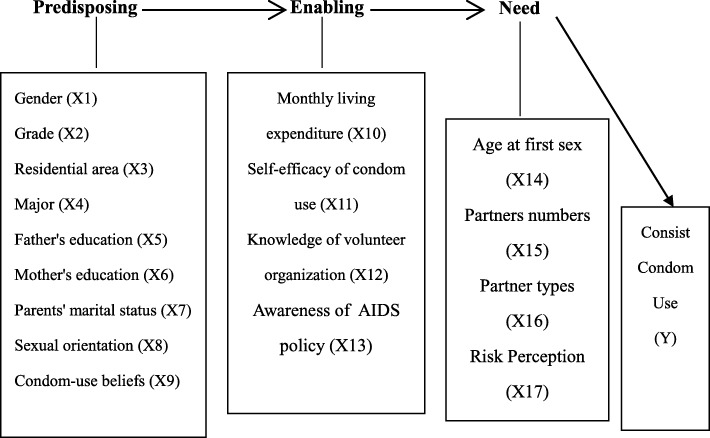
Individual determinants of consistent condom use based on the ABM model

Therefore, mainly informed by the Andersen’s behavioral model and drawing on previous empirical studies on condom use, a conceptual framework was first developed for the present study, illustrated by Fig. 1. Hierarchical logistic regression models were then chosen to estimate their respective contributions of predisposing, enabling and need variables to consistent condom use so as to assess and subsequently to suggest ways to achieve equity in its use among sexually experienced undergraduates in mainland China.

## Hypotheses

As a typical type of discretionary behavior, consistent condom use is hypothesized to be mainly influenced by enabling factors, and inconsistent condom users are mainly heterosexual women and non-heterosexuals, and those with “fewer enabling resources” and “more needs”.

## Methods

### Participants and settings

The data used in this paper were taken from a cross-sectional survey on knowledge, attitude and risk behavior towards HIV/AIDS among college students, carried out from September 10, 2018 to January 9, 2019. The survey involved a total of 12,750 participants distributed across 30 provinces in mainland China (except for Tibet). All statistical analyses in this study were confined to 2054 sexually experienced undergraduates, who must meet the following four inclusion criteria: (a) age 18–25 years (Here it should be pointed out that the age range in this study was widened to 18 to 25 years, given the fact that some students might upgrade from junior college students or enroll later than the compulsory school attendance age); (b) full-time undergraduates currently registered at one university in mainland China; (c) had ever experienced or currently engaged in any form of sex; and (d) answered the questionnaire no later than January 9, 2019.

The protocol was approved by the academic ethics and moral supervision committee from Hubei University of Science and Technology (HUST). Convenience sampling and snowball sampling were used to select the participants. More specifically, on the basis of convenience, diversification and comprehensiveness of majors, college students from HUST were first invited to complete the online questionnaire distributed via the website “www.sojump.com“for credits and even earning the honor of “outstanding volunteers”, and they were also encouraged to recruit future participants from among their acquaintances. In order to get a more geographically diverse sample, Wechat, Sina Weibo, and QQ space were also chosen as the platforms to distribute online questionnaires. After signing electronic informed consent voluntarily, participants completed the questionnaire anonymously and were also promised that all the information they provided would be treated confidentially.

### Design and content of the questionnaire

The self-administrated questionnaire (See Addition file 1) was developed by the Department of Preventive Medicine from HUST and pilot tested with a convenience sample of 50 respondents drawn from the selected population, and has been verified as a valid and reliable measurement tool by our previous large-scale study [37]. This study aimed to identify factors predicting consistent condom use {defined as using condoms during every act of sexual intercourse [14, 15]}, measured by first asking undergraduates whether they had ever experienced or currently engaged in any form of sexual intercourse. Those who admitted to having sexual intercourse were then asked to choose frequency of condom use during sexual intercourse in the past 6 months: never, once in a while, sometimes, every time. Students who reported to use condoms every time when they had sex were categorized as “consistent condom use” and coded as “1”. The remaining respondents were classified as “inconsistent condom use” and coded as “0” (Table 1).

**Table 1:** Bivariable analysis of factors associated with consistent condom use (*n* = 2054)

Variables	Total (*n* = 2054)		Consistent (*n*=1259)		Inconsistent (*n*=795)		χ2	P
	n	%	n	%	n	%		
Gender (X1)								
0 = Female	852	41.5	504	40.0	348	43.8	2.81	0.094
1 = Male	1202	58.5	755	60.0	447	56.2		
Grade (X2)								
0 = Low	934	45.5	547	43.4	387	48.7	5.38*	0.020
1 = High	1120	54.5	712	56.6	408	51.3		
Residential area (X3)								
0 = Rural	1231	59.9	763	60.6	468	58.9	0.61	0.434
1 = Urban	823	40.1	496	39.4	327	41.1		
Major (X4)								
0 = Non-medical	1409	68.6	841	66.8	568	71.4	4.89*	0.027
1 = Medical	645	31.4	418	33.2	227	28.6		
Father’s education (X5)								
0 = Low	1633	79.5	1009	80.1	624	78.5	0.87	0.366
1 = High	421	20.5	250	19.9	171	21.5		
Mother’s education (X6)								
0 = Low	1736	84.5	1075	85.4	661	83.1	1.87	0.172
1 = High	318	15.5	184	14.6	134	16.9		
Parents’ marital status (X7)								
0 = Stable	1682	81.9	1051	83.5	631	79.4	5.54*	0.019
1 = Unstable^b^	372	18.1	208	16.5	164	20.6		
Sexual orientation (X8)								
1 = HeMHeM)	1042	50.7	672	53.4	370	46.5	14.16**	0.003
2 = HeW	709	34.5	427	33.9	282	35.5		
3 = NHeM	160	7.8	83	6.6	77	9.7		
4 = NHeW	143	7.0	77	6.1	66	8.3		
Beliefs about condom use (X9)								
0 = Incorrect	239	11.6	124	9.8	115	14.5	10.10***	0.001
1 = correct	1815	88.4	1135	90.2	680	85.5		
Monthly living expenditure (Yuan RMB^a^) (X10)								
0 = High	344	16.7	194	15.4	150	18.9	4.18*	0.041
1 = Low	1710	83.3	1065	84.6	645	81.1		
Self-efficacy of condom use (X11) (Range = 0–8)								
0 = Low (< 8)	1146	55.8	571	45.4	575	72.3	143.75***	< 0.001
1 = High (=8)	908	44.2	688	54.6	220	27.7		
Knowledge of local volunteer organization (X12)								
0 = No	1064	51.8	606	48.1	458	57.6	17.53***	< 0.001
1 = Yes	990	48.2	653	51.9	337	42.4		
Awareness of the national AIDS policy (X13)								
0 = Unaware	1166	56.8	649	51.5	517	65.0	36.09***	< 0.001
1 = Aware	888	43.2	610	48.5	278	35.0		
Age at first sex (Years) (X14)								
0 = Early	619	30.1	349	27.7	270	34.0	9.02**	0.003
1 = Late	1435	69.9	910	72.3	525	66.0		
The number of sexual partners in the last six months (X15)								
0 = Multiple	178	8.7	80	6.4	98	12.3	21.96***	< 0.001
1 = Single	1876	91.3	1179	93.6	697	87.7		
Partner types (X16)								
0 = Casual	209	10.2	108	8.6	101	12.7	9.08**	0.003
1 = Regular	1845	89.8	1151	91.4	694	87.3		
Risk perception (X17)								
1 = No	1171	57.0	769	61.1	402	50.6	24.87***	< 0.001
2 = Not sure	262	12.8	145	11.5	117	14.7		
3 = Low	544	26.5	309	24.5	235	29.6		
4 = Moderate	23	1.1	11	0.9	12	1.5		
5 = High	54	2.6	25	2.0	29	3.6		

^a^ 1 Yuan RMB =0.1503 US dollars (October 62,017 rate). *p ≤ 0.05; **p ≤ 0.01; ***p ≤ 0.001

^b^ including divorced, separated, remarried, and widowed. Heterosexual men (HeM), heterosexual women (HeW), non-heterosexual men (NHeM), and non-heterosexual women (NHeW)

All the independent variables influencing consistent condom use in this study were classified into predisposing, enabling and need factors based on the Andersen’s behavioral model and also treated as dummy variables because their effects on consistent condom use were found to vary in a non-linear manner (Table 1).

### Predisposing factors

Nine predisposing factors in this study included gender, grade, residential area, major, father’s highest educational level, mother’s highest educational level, parents’ marital status, sexual orientation, and beliefs about condom use. Parents’ marital status was treated as a dummy variable (0 = stable, 1 = unstable, including divorced, separated, remarried, and widowed). Beliefs about condom use was measured by asking the question “Can correct and consistent use of condoms reduce the risk of HIV transmission?”, and three possible responses were provided: Yes, No, or I do not know. In the analysis, “I do not know” and “No” responses were combined and scored as 0 (incorrect), while “Yes” responses were scored as 1 (correct).

### Enabling factors

Four enabling factors in this study included monthly living expenditures, condom use self-efficacy, knowledge of local volunteer organization and awareness of the national AIDS policy. Condom use self-efficacy [14] was measured using an 8-item scale that assessed participants’ ability to negotiate with any sexual partner about the use of condoms in a variety of circumstances (e.g., “Can you use a condom even if sexual partner does not want to?”). For all items above, “unsure” and “no” were combined and scored as “0″, while “yes” was scored as “1″. Scores for the 8 questions were summed to form a self-efficacy index ranging from 0 to 8, with higher scores indicating higher levels of self-efficacy (Cronbach’s alpha = 0.88). In the final analysis, the sum score of this scale was dichotomized at the maximum value of 8 scores into high (1) or low (0) self-efficacy for condom use.

The two variables, including knowledge of local volunteer organization and awareness of the national AIDS policy, were used to measure exposure to the HIV preventive intervention [25, 26]. Since 2003, China has introduced the “Four Frees and One care” policy [19], including free anti-retroviral drugs for those who cannot afford to pay, testing, prevention of mother-to-child transmission, and free schooling of orphans, and care and economic assistance to the households of people living with HIV. Awareness of the national AIDS policy was measured by asking “Do you know the Four Frees and One Care policy?” and three possible responses (Yes/No/Not sure) were presented. In this analysis, “Not sure” and “No” were combined and scored as 0 (Unaware), while “yes” was scored as 1 (Aware). In order to control the HIV epidemic on campus, a growing body of universities have recently established student organizations to distribute free condoms and offer free voluntary HIV counselling and testing (VCT) services. In this study, subjects were classified into two groups (without or with knowledge of local volunteer organization) according to their responses to the Yes/No question “Do you know the student organization in your university to provide free VCT service?”

### Need factors

Three HIV high-risk behaviors, including early sexual initiation, multiple sexual partners and casual sex, were used to reflect the objective and professional evaluation of need for condoms. Multiple and casual sexual partners have been recognized as a risk factor for HIV infection among sexually active male college students in China [2]. Age at first sexual debut is also included in this study because early initiation of sexual activity has been escalating among young students [1, 2] and also because of its well- established relationships with lifetime multiple sexual partners [2, 20, 29]. The question used to derive this variable was, “How old were you when you had sexual intercourse for the first time?” In the final analysis, the variable was grouped into two categories: late or early sexual initiation {defined as having first sexual intercourse under 18 years of age [21]}. The number of sexual partners was measured by asking the question “During the past six months, with how many different people have you had sexual intercourse?” and were classified into two groups [22, 23]: single partner (having only one or no sexual partner) or multiple (i.e. two or more) partners. Partner type was measured by asking the participants to choose their main sexual partners from the four following options: girlfriend/boyfriend, commercial sex worker, one night stand, others (please specify). Participants who reported to have sex only with their girlfriends or boyfriends were categorized as “regular sex” and coded as “1”. The remaining respondents were classified as “casual sex” and coded as “0” (Table 1).

The subjective assessment of one’s need for consistent condom use was indicated by self-perceived risk of HIV infection [16, 18, 23]. College students were asked to estimate their risk of acquiring HIV infection, with five choices provided: no risk, not sure, small, moderate, or great risk [18]. Since only 77 respondents (3.7%) perceived themselves to be at moderate (1.1%) or great risk (2.6%), “moderate or great risk” were combined and “no risk” was chosen as the reference group when performing multivariate logistic regression analyses: (1) Not sure versus no risk; (2) low risk versus no risk; and (3) moderate/high risk versus no risk.

### Data analysis

The data collected via the website “www.sojump.com“were double-cleaned and analyzed independently by two authors using the Chinese version of SPSS 20.0. The statistical analysis was conducted in the following three stages. Firstly, a description was made of all the variables included in the Andersen’s behavioral model. Secondly, variables were screened as candidate predictors for the regression model on the basis of the results of bivariable analysis. For categorical variables the chi-squared test was used, and for ordered categorical variables the chi-squared test for trend was implemented. Finally, those variables that were statistically significant in the bivariate analysis were further entered into multivariate logistic regression model using backward LR method. To identify the relative importance of factors associated with CCU, each set of factors was entered into the logistic regression models in a hierarchical manner [24], with need factors entered first, followed by enabling factors and predisposing factors. This method not only enabled examination of the effects of enabling factors after ruling out the potential confounding effects of need factors, but also made it possible to assess the effects of predisposing factors after considering both need and enabling factors. The change in the − 2 log likelihood associated with predisposing, enabling or need factors entered determined their relative contributions to consistent condom use and how well the model fitted the data when additional variables were added to the model, with smaller values indicating a better fit. Only variables with *P* values less than 0.05 were retained in the final model. The adjusted odds ratio (AOR) and 95% confidence interval (CI) were also reported.

## Results

### Descriptive statistics

**Fig. 2:**
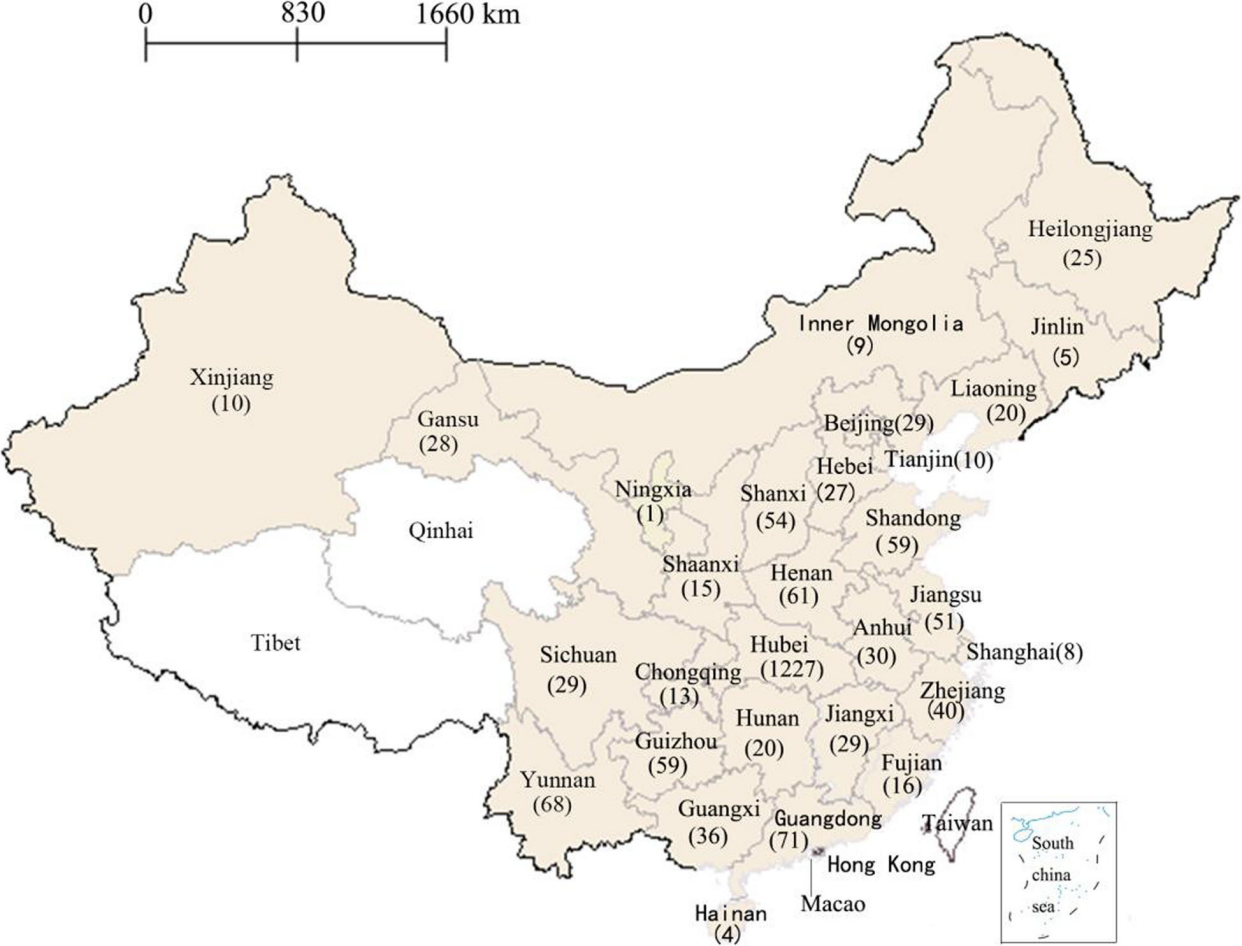
A map displaying the provincial distribution of the 2054 sexually experienced undergraduates was drawn using Supermap iDesktop 8C (2017) and then converted into Microsoft Word format. Excluding Taiwan, Hong Kong, and Macao, China has 31 provincial-level administrations. The exact number in the map indicated that 2054 participants were disproportionately distributed across 29 provinces in mainland China (except for Qinghai and Tibet with white highlighted), and were mainly (1227) recruited from Hubei province

Figure 2 displays the provincial distribution of the 2054 sexually experienced undergraduates. As can be seen from Fig. 2, participants were disproportionately distributed across 29 provinces in mainland China (except for Qinghai Province and Tibet Autonomous Region), and were mainly (59.7%, 1227/2054) recruited from Hubei province. Descriptive statistics for the dependent and independent variables of this study are presented in Table 1. Of the 2054 sexually experienced undergraduates, 61.3% (95% CI:59.2–63.4%) reported to use condoms consistently during every sexual encounter. The predisposing variables revealed that slightly more than half of the sample was male (58.5%), had completed two or more years of college (54.5%), and came from rural areas (59.9%). Nearly one third of the sample (31.4%) was medical students. Nearly one-fifth of the participants reported that their father (20.5%) or mother (15.5%) had obtained a college degree or above, and their parents had kept unstable marital relationships (18.1%). To our surprise, 14.8% identified themselves as non-heterosexual men (7.8%) and women (7.0%), and 11.6% did not believe that correct and consistent use of condoms can reduce the risk of HIV transmission.

In terms of enabling variables, 16.7% spent each month more than 2 thousand Yuan [1Yuan RMB = 0.148951094 US dollars (March 25, 2019 rate)] on food and other basic commodities. Approximately half of students were knowledgeable about local volunteer organization (48.2%) and the national AIDS policy (43.2%), and were wholly confident of negotiating condom use with their sexual partners (44.2%).

In terms of need-for-condom factors, nearly one-third (30.1%) of respondents had their first sexual intercourse under 18 years of age, and around one-tenth of sexually experienced college students admitted to having multiple sexual partners in the past 6 months (8.7%) or having sex with casual partners (10.2%). However, only 3.7% perceived themselves to be at moderate/high risk of HIV infection.

### Bivariable analysis

The results of the bivariable analysis were shown in Table 1. Thirteen independent variables, as indicated in Table 1, were found to be significantly associated with consistent condom use. More specifically, those who used condoms consistently tended to be high-grade, medical students, their parents remaining in a stable marital relationship, heterosexual women and non-heterosexuals, having a correct knowledge of condom use, lower living expenditure, high levels of self efficacy, more knowledgeable of the national AIDS policy and local volunteer organization, late sexual initiation, single sexual partner, regular sex, and self- perceived lower risk of HIV infection. No statistically significant differences were reported between consistent and inconsistent condom users in grade, residential area, father’s education, and mother’s education.

### Hierarchical logistic regression

The value of − 2 log likelihood was significantly reduced when each set of factors (i.e., four need factors, four enabling factors, and five predisposing factors) was introduced into the logistic regression model, with enabling factors producing the largest reduction. These results indicated that consistent condom use (CCU) was inequitably distributed, since it was mainly influenced by enabling resources rather than by need factors of sexually experienced college students.

Model 1 included only the four need factors and indicated that Age at first sex, Partner number and Risk perception were significantly associated with consistent condom use (CCU). When the four enabling factors were added to the model (Model 2), Condom use self-efficacy and Awareness of the national AIDS policy were statistically significant, while Risk perception lost its significance. The final model, Model 3, had the best overall model fit as indicated by the − 2 Log Likelihood. When combining all the 13 significant variables (i.e., four need factors, four enabling factors, and five predisposing factors) into the final logistic regression model (Model 3) and adjusting for potential confounding factors, only one predisposing factor (Sexual orientation), two enabling factors (Condom use self-efficacy and Awareness of the national AIDS policy) and two need factors (Age at first sex and Partner number) were significantly associated with CCU.

Compared with heterosexual men, heterosexual women (AOR = 0.78, 95% CI: 0.64–0.96), non-heterosexuals men (AOR = 0.64, 95% CI:0.45–0.92) and women (AOR = 0.68, 95% CI:0.47–0.99) were less prone to using condoms consistently. Those with more enabling resources (i.e., higher levels of self-efficacy for condom use and being knowledgeable of the national AIDS policy) and those with lower need for condoms (i.e., late sexual initiation and single sexual partner) were more likely to be consistent condom users, when compared with their respective counterparts. More specifically, having higher levels of self-efficacy increased one’s odds of CCU by 1.86 times (95% CI:2.35–3.49). The odds of CCU for those being knowledgeable about the national AIDS policy were 1.50 times of those being ignorant of the policy (95% CI:1.23–1.82). Those who initiated their first sexual intercourse at age 18 or older were 1.34 times (95% CI:1.09–1.64) more likely to use condoms consistently, compared with those who initiated their first sexual intercourse under the age of 18 years. Similarly, compared to those having multiple sexual partners, the likelihood of using condoms consistently were 1.68 times (95% CI:1.21–2.33) for those with only one single partner (See Model 3 in Table 2).

**Table 2:** Logistic regression analysis of factors associated with consistent condom use

	Model 1 ^a^		Model 2^b^		Model 3^c^	
	AOR	95% CI	AOR	95% CI	AOR	95% CI
Block 1: Need factors						
Age at first sex						
0 = Early, 1 = Late	1.28^*^	1.05–1.55	1.36^**^	1.11–1.66	1.34^**^	1.09–1.64
Partners numbers						
0 = Multiple, 1 = Single	1.87^***^***	1.36–2.57	1.73^***^	1.25–2.40	1.68^**^	1.21–2.33
Risk perception (ref = No risk)						
Not Sure	0.66^**^	0.50–0.87				
Low risk	0.68^***^	0.55–0.84				
Moderate/high risk	0.55^*^	0.34–0.89				
Block 2:Enabling factors						
Self-efficacy of condom use						
0 = Low, 1 = High			2.82^***^	2.32–3.43	2.86^***^	2.35–3.49
Awareness of the national AIDS policy						
0 = Unaware, 1 = Aware			1.52^***^	1.25–1.85	1.50^***^	1.23–1.82
Block 3: Predisposing factors						
Sexual orientation (ref = Heterosexual men)						
Heterosexual women					0.78^*^	0.64–0.96
Non-heterosexual men					0.64^*^	0.45–0.92
Non-heterosexual Women					0.68^*^	0.47–0.99
-2 Log likelihood	2693.75		2558.69		2547.70	
Change of −2 log likelihood, d.f.	48.25, 5		130.10, 2		15.20, 3	
*P* value	< 0.001		< 0.001		< 0.001	

d.f., degree of freedom; AOR, adjusted odds ratio; CI, confidence interval

^a^Model 1: Age at first sex, Partner number, Partner type and Risk perception;

^b^Model 2: Age at first sex, Partner number, Partner type, Risk perception, Monthly living expenditure, Self- efficacy of condom use, Knowledge of local volunteer organization, and Awareness of the national AIDS policy;

^c^Model 3: Age at first sex, Partner number, Partner type, Risk perception, Monthly living expenditure, Self- efficacy of condom use, Knowledge of local volunteer organization, Awareness of the national AIDS policy, Grade, Parents’ marital status, Major, Sexual orientation and Beliefs about condom use

## Discussion

### Main findings of this study

In our sample, only 61.3% reported to use condoms consistently during every sexual encounter. This underscores the urgent need for implementing the “100% condom use program (CUP)” to reduce the vulnerability of unmarried sexually active college students to unwanted pregnancy, HIV infection and other STDs [2, 3]. Based on the Andersen’s behavioral model, hierarchical logistic regression revealed that CCU was inequitably distributed since its use was primarily influenced by enabling factors. Heterosexual women, non-heterosexual men and women, and those with less enabling resources (i.e., lower self-efficacy for condom use and being ignorant of the national AIDS policy) and with higher need for condoms (i.e., early sex initiation and multiple sexual partners) were less likely to be consistent condom users.

### Comparisons with previous studies

The concentration of inconsistent condom use among sexually experienced undergraduates in mainland China with earlier initiation of sexual intercourse and multiple sexual partners is especially notable. The findings from this study support previous research [2, 26, 29] that have found that individuals with early sexual debut were more likely to engage in risky sexual behaviors such as multiple sexual partners and inconsistent condom use and thus contributing to increased risks of unintended pregnancies, HIV infection and other STDs.

Self-efficacy was defined as an individual’s perception of his or her ability to successfully engage in a selected health behavior. Consistent with previous research [7, 8, 11, 14, 16, 17, 26], perceived self-efficacy of condom use was identified as a significant enabling factor that assists respondents in using condoms consistently. This could be due to the fact that individuals with higher condom use self-efficacy emerged as perceiving less difficulty in making condom requests and actually using condoms more consistently, compared with those reporting lower condom use self-efficacy.

Our study indicated that individuals who were knowledgeable about the national AIDS policy were more likely to use condoms consistently. This finding is not surprising as it fits the general pattern of positive intervention [25, 26]. Those with knowledge about the national AIDS policy often gain a better insight into symptoms of HIV disease, know more about the availability of free condoms and use this information more effectively to access to sexual health services.

Consistent with a previous study on the relationship between treatment optimism, safer sex burnout and consistent condom use [33], sexual orientation was found to be statistically significant, with those identifying themselves as heterosexual women, non-heterosexual men and non-heterosexual women less likely to be consistent condom users than heterosexual men (AOR = 0.78, 0.64 and 0.68, respectively). The increased vulnerability of heterosexual females was mainly attributable to social gender roles and gender differences [26, 28], especially in China where women had less power to negotiate safe sex [9] and male partners were already identified as a major barrier to condom use for those women who desired to use this male-controlled device [28]. The lower rates of consistent condom use reported among non- heterosexuals in this study might be due to the fact condoms in China were mainly used to prevent pregnancy, rather than for STDs prevention [11, 32], or because non- heterosexuals, especially MSM would choose other safer sex practices such as PrEP, serosorting and strategic positioning (commonly called “seroadaptive behaviors”) as alternative strategies to consistent condom use [33–35]. For example, one study in Taiwan demonstrated that serosorting had emerged as a harm reduction approach for HIV-positive MSM to reduce the risk of HIV transmission with unprotected sex [35].

### Limitations

Several limitations should be considered when interpreting the findings of this study. First, since this study relied on a cross-sectional design to measure consistent condom use and its influencing factors simultaneously, it was difficult to infer a cause and effect relationship observed between these variables. Second, despite considerable efforts to obtain a more geographically diverse sample, we mainly adopted the method of convenience sampling and snowball sampling to select respondents, thus leading to the over-representation of respondents from Hubei Province and limiting generalizability or external validity of the results. Third, this survey relied on participants’ self-reported condom use which may suffer from social desirability bias, thus leading to overestimates of condom use [30]. For example, using prostate specific antigen as a gold standard biomarker, Liu and colleagues [31] have found that 26–46% of mid-aged female sex workers in China over-reported condom use with all sex partners including husbands, boyfriends or clients. However, an anonymous and confidential online self-administered survey, rather than the face-to-face interviewer- administered questionnaire, was carried out to minimize potential biases.

### Implications of the study

In spite of the stated limitations, the findings from our study have several implications for the design and implementation of HIV prevention interventions on college campuses. To the best of our knowledge, ours is the first to employ the Andersen’s behavioral model as theoretical framework, combined with hierarchical regression models, to examine equitable distribution of consistent condom use among sexually experienced undergraduates in mainland China. Our findings suggested that equal use of condoms for equal need-for-condoms had not been achieved and that inconsistent condom users were mainly those with higher need for condoms (i.e., earlier initiation of sexual intercourse and multiple sexual partner) and those with less enabling resources (i.e., lower condom use self-efficacy and being ignorant of the national AIDS policy. Furthermore, heterosexual women, non-heterosexual men and women were less likely to use condoms consistently than heterosexual men. Consequently, four main types of intervention aimed at reducing inequities in consistent condom use are recommended.

1. Target students with higher need for condoms: A tendency for early entry to sexual initiation to have multiple sexual partners and subsequently to use condoms inconsistently would be labeled as “equitable” and “immutable”. Therefore, the “100% Condom Use Program” should be immediately promoted among students exhibiting these characteristics, while our long-term goals should be set to delay sexual intercourse and decrease the number of sexual partners. In addition, our results also indicated that nearly one-third of respondents had initiated their first sex below the age of 18 years (the minimum age requirement for admission to a university). Therefore, sex education should be started earlier and more sexual and reproductive health services should be provided to help adolescents to make wise sexual decisions. Fortunately, the Chinese government has already realized the urgency of this issue and began to take action to solve it.
2. Enhance condom-use self-efficacy: The enabling variable which has the strongest effects on CCU is self-efficacy for condom use. Its effects are judged “mutable” and “inequitable”. In order to promote consistent use of condoms and also achieve its equity, the first and foremost intervention is to improve their skills for negotiation of condom use. Also, the intervention should focus on the individuals’ perceived barriers to negotiating with sexual partners about condom use and develop appropriate strategies to make these individuals feel at ease with the negotiating process [14].
3. Improve awareness of the national AIDS policy: Awareness of the national AIDS policy was judged “mutable” and “inequitable”. Consequently, continued effort should be made to recruit and train student volunteers to propagate the national AIDS policy (i.e., “Four Frees and One care” policy) and expand sex education program to make free condoms to be available for students.
4. Focus on heterosexual women and non-heterosexuals: Heterosexual women and non-heterosexuals, especially non-heterosexual men with decreased odds of consistent condom use would be labeled as “inequitable” and “immutable”. It cannot be denied that consistent and correct condom use remains the goal of preventing unintended pregnancies, HIV infection and other STDs at the population level [14]. However, due to their vulnerability and difficulty in using condoms consistently, future prevention efforts should include combine multiple intervention strategies [36] and continue to be focused on high-risk groups, especially MSM. For example, interventions targeting at MSM for HIV prevention should include but not be limited to encouraging abstinence, routine testing and treatment of STDs, and taking PrEP.

## Conclusions

While other studies have described consistent condom use as an infrequently adopted protective behavior, ours is the first to examine equitable distribution of consistent condom use through the application of hierarchical logistic regression in conjunction with the Andersen’s behavioral model both in the general population and among most-at-risk populations. Our findings suggested the overall level of consistent condom use has remained low among sexually experienced undergraduates in China. Furthermore, consistent condom use was inequitably allocated, since enabling factors exerted greater effects than predisposing and need variables. Inconsistent condom users were mainly heterosexual women and non-heterosexuals, and those with “fewer enabling resources” and “more needs”. In order to increase consistency of condom use and simultaneously reduce the remaining inequities, a comprehensive intervention measure should be taken to target heterosexual women and non- heterosexual undergraduates and those with higher need for condoms, improve their condom use self-efficacy and raise their awareness of the national AIDS policy.

## Abbreviations

ABM: Andersen’s behavioral model
AOR: Adjusted odds ratio
CCU: Consistent condom use
CI: Confidence Interval
HIV: Human Immunodeficiency Syndrome
HUST: Hubei University of Science and Technology
SPSS: Statistical package for Social
STD: Sexually transmitted disease
